# The Interaction Between Accessions and Fruit Maturity Stages in *Mimusops zeyheri* and Its Impact on Postharvest Quality and Nutritional Composition

**DOI:** 10.1002/fsn3.70015

**Published:** 2025-02-10

**Authors:** Kamogelo P. Teffo, Semakaleng Mpai, Ashwell R. Ndhlala, Phatu W. Mashela

**Affiliations:** ^1^ Green Biotechnologies Research Centre of Excellence, Department of Plant Production, Soil Science and Agricultural Engineering University of Limpopo Sovenga South Africa

**Keywords:** accession variations, food security, indigenous fruit, *Mimusops zeyheri*, nutritional content, Transvaal red milkwood

## Abstract

*Mimusops zeyheri* Sond is an undervalued indigenous fruit tree with fruits that are consumed as a health snack in rural communities aross Sub‐Saharan Africa. This study aimed to assess the interaction effect of five accessions of *M. zeyheri* and four fruit maturity stages on some quality and nutritional compositions. Fruits of five *M. zeyheri* accessions were grouped into four maturity stages for analysis of fruit size, fruit firmness, total soluble solids, titratable acidity, proximate analysis, and amino acids. Accession 6E consistently had the highest size (27.62 mm), while accession 3L (6.40 kg) had the highest fruit firmness. Accession M7 displayed the highest TA (3.20%) at dark green unripe stage (T1). Highest moisture content and protein percentage were recorded in accession HY at T1. This changes were in concomitant to an increase in moisture content and a decrease in ash and protein content. Accessions M7 at T1 to T4 maturity stage exhibited the highest essential amino acids including histidine and threonine, as well as Ca, Mg, and Na. Accessions 3E and 6E at T2 and T3 maturity stage exhibited the highest P, Fe, Zn, and Mn. These findings highlight the variability of physicochemical and nutritional compositions among different *M. zeyheri* accessions at varying stages of fruit maturity.

## Graphical abstract

The study examines the variation in quality and physicochemical properties of five *Mimusops zeyheri* accessions at four maturity stages. Significant findings include an increase in total soluble solids and moisture content alongside decreased firmness and acidity. Variations in proximate compositions across accessions highlight *M. zeyheri* accessions potential as a nutritious fruit source, suitable for fresh consumption or food supplementation, promoting food security and supporting rural development.

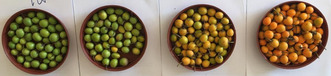

## 1 Introduction

In South Africa, indigenous fruits constitute an important part in the food basket of the rural and/or township households (Mkhathini et al. 2017; Akinola et al. 2020). *Mimusops zeyheri* Sond commonly known as Transvaal red milkwood or *Mmupudu* (in *Sepedi*) is a member of the Sapotaceae Family and it is described as undervalued and unappreciated indigenous fruit tree (Omotayo et al. 2020). Its fruits are consumed raw as a healthful snack in rural communities across Sub‐Saharan Africa (Hankey 2005; Matlala et al. 2024). It is predominantly high in vitamin C (Mngadi, Moodley, and Jonnalagadda 2017). *M. zeyheri* is a perennial evergreen tree that yield oval‐shaped fruits with pointed tips which turn from green to yellow or orange during ripening (Mashela and Mollel 2001; Satekge et al. 2022). During its development stage, the fruits undergo convention of the green pigmentation chlorophyll and starch to carotenoids and sugars, components that improve the taste of the fruits (Kopsell et al. 2005). Five accessions of *M. zeyheri* trees have been identified and collected around Southern African region including South Africa, Zimbabwe, and Botswana (Mashela and Mollel 2001).

Currently, there rarely are studies that have assessed an interaction effect between accessions variation and fruit maturity stages on postharvest quality, physicochemical, and nutritional compositions of *M. zeyheri* and it remains unknown. The recognition and incorporation of indigenous fruits into mainstream food system could possibly contribute to the reduction and eradication of food poverty (Sibiya, Kayitesi, and Moteetee 2021). In light of this, indigenous fruits are better suited candidates due to their adaptability to marginal growth conditions, ease of accessibility, and their high nutritional values. Analysis of the nutritional content of edible indigenous fruits at different fruit maturity stages could provide information on the quality, and evidence‐based information on their potential as food base. Such knowledge contributes to the commercialization, utilization, promotion, and future research (Omotayo et al. 2020). In addition, indigenous fruits are highly and widely recommended given that are sources of mineral nutrients and amino acids, which confer health benefits and reduce risks of diseases risks (Ngadze et al. 2017).

Despite the well‐documented ethnobotanical literature in South Africa (Omotayo et al. 2020), published literature show very little information on the quality, physicochemical, and nutritional compositions of *M. zeyheri* fruits at different fruit maturity stage is still not available. In this study, the aim was to determine the interactive effect of five accessions of *M. zeyheri* harvested at four different fruit maturity stages on quality, physiochemical, and nutritional compositions.

## 2 Materials and Methods

### 2.1 Study Location, Treatments, and Experiment Design

**FIGURE 1:**
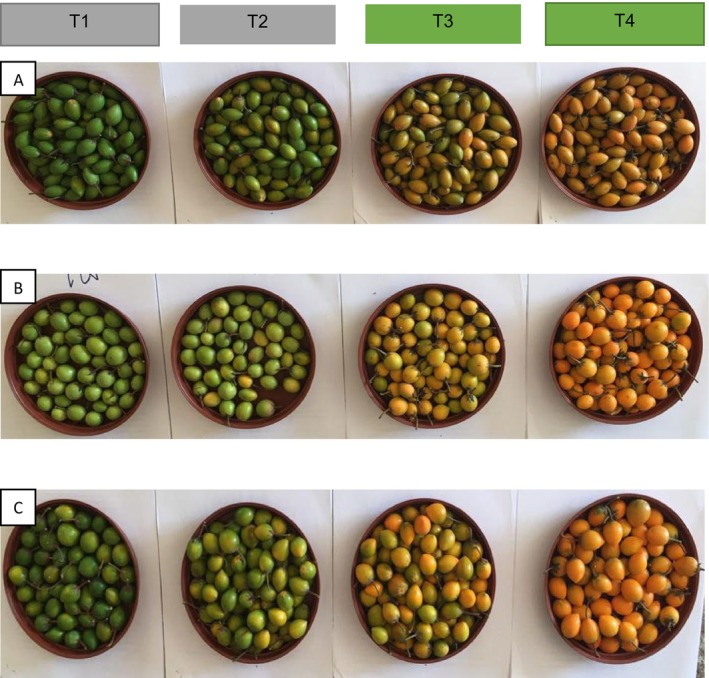
The schematic version of five accessions *M. zeyheri*, represented by letters (A: 6E; B: M7; and C: HY) at four fruit maturity stages (T1: Green; T2: Breaker; T3: Pale yellow; and T4: Yellow fruit).

Fruits of *M. zeyheri* were harvested from domesticated trees, planted at the Green Biotechnologies Research Centre of Excellence, University of Limpopo, South Africa (23°5300100 S, 29°4400150 E). These plants were different accessions based on location of origin. The area receives minimum average rainfall of 500 mm and the site consists of hutton soil type. Moreover, summer temperatures range from 28°C to 38°C while winter temperature range from 5°C to 17°C. Fruits were harvested randomly from trees of five accessions namely: 6E, M7, 3E, HY, and 3L. The experimental designed followed a 5 × 4 factorial design for the interactive between five accessions (6E, M7, 3E, HY, and 3L) and four fruit maturity stages: dark green (T1), breaker (T2), and pale yellow (T3) and dark yellow/orange (T4) as shown in Figure 1A–C. The experiment was laid in a split plot design with four replications. For each accession and maturity stage, consist of 20 fruits per replication (*n* = 20).

### 2.2 Harvesting and Sample Preparation

Fruits were harvested in the month of November in 2022. Stages of fruit maturity were determined according to Ndou et al. (2019), whereby visual skin color was used as a key index for classification. Four fruit maturity stages were considered as described above (dark green [T1], breaker [T2], and pale yellow [T3], and dark yellow/orange [T4]). The pulp and peel of the fruits were carefully separated from the seed and mixed using a mortar and pestle. The samples were oven‐dried at 40°C until a constant weight was achieved and grounded into powder prior to the analysis.

### 2.3 Determination of Quality and Physicochemical Attributes

The length and width of each fruit were measured using a vernier caliper (Mitutoyo 500, South Africa) and expressed in mm. Fruit firmness was determined using a handheld firmness penetrometer (Effigi 11 mm Prob, T.R. Turoni Srl. Italy). The fruits were crushed using pestle and mortar and the collected juice was tested for TSS using a digital refractometer (121, Yagami International Ltd., Tokyo, Japan). Few drops were placed on the prism of the refractometer to allow for reading measurements (Pila, Gol, and Rao 2010). Total soluble solids (TSSs) of the fruits were expressed in Brix° (%).

Titratable acidity (TA) determined by peeling fruits, 10 g of the pulp, for each accession and fruit maturity stage, was weighed and homogenized with 40 mL of 0.1 N sodium hydroxide (NaOH). Thereafter, the mixtures were centrifuged at 2, 414. 9 g for 5 min at 2, 414. 9 g and the supernatant 10 ml were recovered added into conical flasks for titration using drops of phenolphthalein indicator. TA was calculated using the following formula:

$$\%\text{malic acid}=\text{volume}\;\left(V\right)\times N\times \text{equivalent factor}/\mathrm{mL}$$

where v represents the volume titrated, N indicates the NaOH normality, and mL represents milliliters of juice. Equivalent factor of the predominant malic acid was 0.067.

TSS/TA ratio was calculated by dividing the TSS value of each fruit juice sample by that of the percentage of TA (Brix°/Acid), respectively (Rooban et al. 2016). The color of the fruits was analyzed using a Minolta CR‐400 Chroma meter (Minolta, Osaka, Japan), which uses the Munsell color system specified for three dimensions such as lightness, hue angle and chromaticity. The color value L* indicates (0 = black and 100 = white), a* represent redness and b* indicates yellowness of the fruit and hue angle were displayed automatically and recorded (Chepngeno et al. 2016).

### 2.4 Determination of Proximate Compositions

Moisture content was determined in accordance with AOAC standard method (AOAC 2000) by comparing the initial weight of fresh samples with their weight after 72 h of drying at 30°C. Dry matter content (%) was calculated by heating fruits at 30°C for 72 h until a constant weight was achieved, with the weight loss used to compute moisture and dry matter content. Ash content, representing the residual matter post‐oxidation of organic components, was determined using the AACC (1999) method. Crucibles were pre‐dried at 100°C for 5 h, then each 2 g sample was weighed into crucibles. The samples in crucibles were combusted on a tripod, followed by a 5‐h muffle furnace treatment at 550°C. After cooling in a desiccator to room temperature, the samples were weighed.

The AOAC (1990) micro Kjeldahl method was employed to determine protein content, wherein 2 g of each sample was placed in a heating tube with 10 mL of concentrated sulfuric acid (H_2_SO_4_) and one selenium catalyst tablet. The mixture was heated in a fume closet, and the resulting digest was transferred to a 100 mL volumetric flask and diluted with distilled water. Subsequently, 10 mL of the digest was combined with an equal volume of 45% NaOH and subjected to Kjeldahl distillation. The distillate was then mixed with 4% boric acid and indicator. After collecting 50 mL of distillate, titration was performed for both samples, and the average value was determined. Using a protein conversion factor of 6.25, the nitrogen content was calculated and expressed as a percentage of protein (Mokgalabone, Mpai, and Ndhlala 2023).

### 2.5 Determination of Amino Acids

Following the procedures outlined by Mpai et al. (2018), the dried peel and pulp samples were processed for analysis of amino acids using an Amino Acid Analysis Application Solution (AccQ Tag) derivatization kit. The analysis of essential amino acids, including histidine (His), threonine (Thr), lysine (Lys), valine (Val), leucine (Leu), isoleucine (Ile), phenylalanine (Phe), and nonessential amino acids including arginine (Arg), serine (Ser), glycine (Gly), aspartic acid (Asp), glutamine acid (Glu), alanine (Ala), proline (Pro), and tyrosine (Tyr) was performed using the ultra‐performance liquid chromatography analysis following the methods described by Mpai et al. (2018).

### 2.6 Determination of Mineral Compositions

A total of 10 g powder of dried peel and pulp of fruit samples were digested in 40 mL of 4% nitric acid before the mixture was completely wetted by spinning the container on a vortex. After the samples had been magnetically agitated, they were placed in a 95°C water bath. After a 90 min incubation on the water bath, the samples was cooled to ambient temperature, filtered, and decanted into 50 mL tubes that were foil wrapped before being subjected to analysis of metal elements (phosphorus [P], potassium [K], calcium [Ca], magnesium [mg], sodium [Na], Iron [Fe], manganese [Mn], and zinc [Zn]) using inductively coupled plasma‐optical emission spectrometry (ICPE‐9000). The analysis's circumstances and the growth of the mineral standard curve mirrored those mentioned by Mokgalabone, Mpai, and Ndhlala (2023).

### 2.7 Statistical Analysis

Data are presented as means ± standard error after being subjected to statistical analysis using GenStat 18th version statistical package (VSN International, Hempstead, UK). The graph Pad Prism 5 was used to plot the graphs. The mean separation for significant variation was achieved using a Duncan at the significance level of 5%.

## 3 Results and Discussion

### 3.1 Variation of Five Accessions and Four Fruit Maturity Stages on Quality and Physicochemical Parameters

**FIGURE 2:**
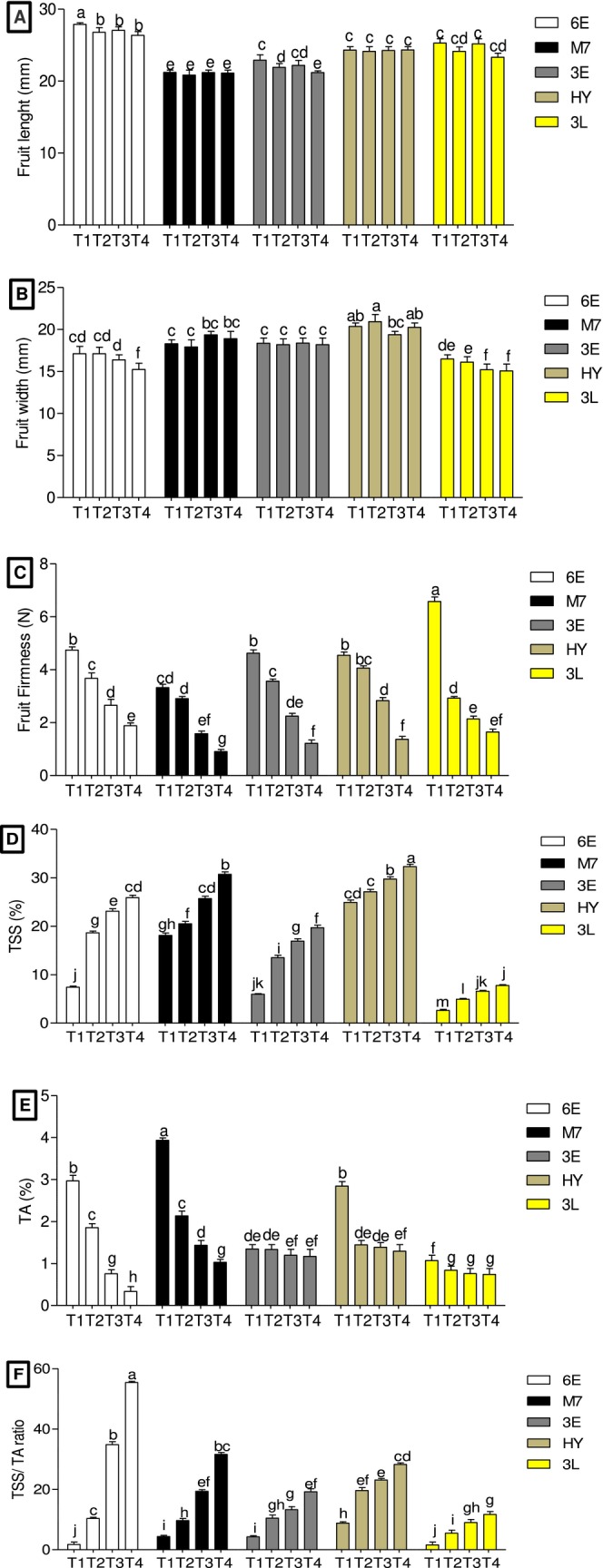
(A) Fruit length, (B) Fruit width, (C) Fruit firmness, (D) TSS, (E) TA, and (F) TSS/TA ratio. The effect of quality and physicochemical attributes at four fruit maturity stages (T1–T4) in five accessions of *M. zeyheri*. Fruit maturity stages (T1: Green; T2: Breaker; T3: Pale yellow; and T4: Yellow fruits). Bars (±SD) with different letters are significantly different (*p* > 0.05) according to Duncan's multiple range test.

Figure 2A,B illustrated five accessions of *M. zeyheri* harvested at four maturity stages on fruit size. The results revealed significant (*p >* 0.05) variation such that accession 6E consistently had the highest fruit length from T1 (Green) (25.85 mm) to T4 (Dark yellow) (27.63 mm) of maturity stages, respectively, while the other accessions (3E, HY, and 3L) exhibited similar and intermediate fruit lengths, all higher than accession M7, which had the lowest fruit length. In terms of fruit width, accessions 6E, M7, HY, and 3E showed similarity, but all had wider fruits than accession 3L T1 (Green) and T4 (Dark yellow) ranging between 15.99 and 14.25 mm regardless of the maturity stage. These findings indicate heterogeneity in fruit size and maturity stages. The results also demonstrate an accumulative growth pattern during fruit development, with significant increases in length and width at T1 (Green) maturity stage compared to T3 (Pale yellow) and T4 (Dark yellow) in all the accessions. This pattern aligns with previous studies on deciduous fruits like mango Muiruri et al. (2022). The differences in length and width may be attributed to genetically make‐up of the fruit, which contributes to variation of cell division and expansion patterns during the initial growth stages of the fruits Ndou et al. (2019). The study found significant variations in fruit firmness, with the highest mean values observed at the T1 (Green) maturity stage and the lowest at the T4 (Dark yellow) stage across all accessions (Figure 2C). Accession 3L exhibited the highest fruit firmness at 6.40 kg at T1 (Green), while accession M7 had the lowest at 0.83 kg T4 (Dark yellow). Similar variations in fruit firmness have been reported in previous study of Rooban et al. (2016). The results also revealed that as fruits progressed from T1 (Green) to T4 (Dark yellow) maturity stages, their firmness decreased, which is a critical factor affecting fruit quality and postharvest shelf life (Oluwaseun, David, and Issa 2013). This reduction in firmness is associated with the ripening process, where physiological changes in cell wall structure, driven by enzymes and polysaccharides, lead to fruit softening (Atkinson and Macrae 2007; Tian and Xu 2023). Conversely, softer fruits at the T4 (Dark yellow) maturity stage are suitable for producing fruit juices for dried fruits. The study revealed significant (*p >* 0.05) variations in TSS content, with a consistent increase from T1 (Green) to T4 (Dark yellow) maturity stages across all accessions. Accession HY displayed the highest mean TSS values at T1 (Green) to T4 (Dark yellow) maturity stages (24.40–32.60 Brix°), while accession 3L had the lowest mean TSS values (2.40–7.60 Brix°), respectively (Figure 2D). These findings align with previous research by Wu et al. (2005), Rooban et al. (2016), and Ndou et al. (2019), which noted an increase in TSS content as fruits ripened and became ready to eat. TSS content is considered a crucial physiochemical attribute of fruit, as it determines the concentration of soluble solids in liquids, influencing the taste and sweetness level of the fruit, as highlighted by Kusumiyati et al. (2022).

The TA of fruits from selected *M. zeyheri* accessions at different maturity stages, revealing significant variation. Generally, there was a gradual decrease in TA as the fruits ripened, as illustrated in Figure 2E. Accession M7 displayed the highest TA at the T1 (Green) maturity stage (3.88%), surpassing all other accessions, while accession 3L consistently had the lowest TA values across (Green) to T4 (Dark yellow) maturity stages (0.94%–0.64%). Notably, fruits at the T1 (Green) maturity stage exhibited higher TA compared to those at the T4 (Dark yellow) maturity stage. Among the accessions, 6E at the T4 (Dark yellow) maturity stage had the lowest mean TA value (0.22%) compared to its counterparts. This trend of decreasing TA content during fruit ripening is consistent with findings in other fruits, such as blueberries, mango, and cashew apple (Rooban et al. 2016). TA reflects the concentration of acids in fruits, and its decline during ripening is attributed to the conversion of starch into sugars, reducing acidity (Zhang et al. 2021). The TSS/TA ratio in fruits of *M. zeyheri* from five different accessions at various maturity stages, revealing significant variation. The TSS/TA ratio showed a significant increase as the fruits advanced in maturity, with the highest mean value observed at the T4 (Dark yellow) maturity stage for all accessions, as depicted in Figure 2F. Accession HY exhibited the highest mean TSS/TA ratio values at T1 (Green) to T3 (Pale yellow) maturity stages (9.27%–23.61%). Conversely, accession 3L consistently had the lowest TSS/TA ratio values at T1 (Green) to T4 (Dark yellow) maturity stages (ranging from 2.55% to 12.67%). Notably, at the T4 (Dark yellow) maturity stage, accession 6E had the highest TSS/TA ratio (56.78%), in line with findings by Ndou et al. (2019). The TSS/TA ratio's increase with fruit maturity stages aligns with prior research and is linked to the rise in TSS values with fruit ripening, coupled with decreasing TA values from T1 (Green) to T4 (Dark yellow) maturity stages, as shown in Figure 2F. This trend is attributed to gluconeogenesis, the hydrolysis of polysaccharides like starch, reduced acidity, and the presence of sugar and organic acids during the maturation period, as explained by Ndou et al. (2019). Consequently, the TSS/TA ratio can serve as a valuable indicator to determine the maturity stages of fruits from different accessions.

The variation in Hunter color parameters (a*, L*, and h° angle) in fruits from five *M. zeyheri* accessions at different maturity stages, with significant differences observed (Table 1). The mean values of Hunter a* varied across accessions and maturity stages, with accession 3L values increasing from T1 (Green) to T4 (Dark yellow) maturity stages. Notably, accession HY had the highest Hunter a* value at T1 (Green) maturity stage (4.91), accession 3E at T2 (Breaker) (4.06), and accession M7 at T3 (Pale yellow) (5.36) and T4 (Dark yellow) (5.49) maturity stages. This change in fruit color, transitioning from green to yellowish, is associated with the breakdown of chlorophyll and the development of carotenoids on the fruit peel or flesh, as noted by Nambi et al. (2016). The mean values of Hunter L* were highest at T1 (Green) and lowest at T4 (Dark yellow) maturity stages across all accessions. Accession 6E had the highest values at T1 (Green) and T3 (Dark yellow) maturity stages, while accession M7 exhibited the highest values at T2 (Breaker) and T4 (Dark yellow) maturity stages. The h° angle showed increased mean values at T1 (Green) maturity stage and the least at T4 (Dark yellow) as the fruit ripened. Accession 6E had the highest value at T1 (Green), accession 3L at T2 (Breaker) and T3 (Pale yellow), and accession 3E at T4 (Dark yellow). These changes in Hunter color parameters indicate a shift from green to dark yellow fruit color as the fruits advanced through maturity stages, a phenomenon also observed in papaya by Zuhair et al. (2013). The increased Hunter a* and b* values further corroborate the color transition from green to dark yellow with maturation, in line with findings from Kim et al. (2013).

**TABLE 1:** List of color change coordinates of five accessions of *Mimusops zeyheri* fruits at four fruit maturity stages.

Accessions	Fruit maturity stages	Color change coordinates		
		a*	L*	h° angle
6E	T1 (Green)	3.35 ± 0.28^ij^	63.00 ± 1.09^a^	73.25 ± 1.31^a^
	T2 (Breaker)	3.28 ± 0.06^i^	57.82 ± 0.52^cd^	65.29 ± 1.01^e^
	T3 (Pale yellow)	4.11 ± 0.05^f^	62.02 ± 0.35^ab^	70.25 ± 0.57^abc^
	T4 (Dark yellow)	5.05 ± 0.48^c^	57.55 ± 2.07^d^	58.92 ± 3.28^g^
M7	T1 (Green)	4.15 ± 0.57^f^	59.77 ± 1.77^cd^	61.84 ± 2.10^ef^
	T2 (Breaker)	3.87 ± 0.60^hi^	62.97 ± 3.28^ab^	70.08 ± 1.05^c^
	T3 (Pale yellow)	5.36 ± 0.32^b^	57.36 ± 2.93^de^	57.97 ± 3.62^h^
	T4 (Dark yellow)	5.49 ± 0.40^a^	58.91 ± 3.37^d^	59.72 ± 4.10^def^
3E	T1 (Green)	3.45 ± 0.33^def^	60.27 ± 2.15^ab^	67.94 ± 4.45^d^
	T2 (Breaker)	4.06 ± 0.14^fg^	59.69 ± 0.62^c^	67.37 ± 1.25^e^
	T3 (Pale yellow)	4.53 ± 0.34^de^	61.12 ± 2.79^ab^	67.60 ± 2.28^de^
	T4 (Dark yellow)	4.54 ± 0.54^de^	57.82 ± 2.79^cd^	62.78 ± 3.72^ef^
HY	T1 (Green)	4.91 ± 0.56^d^	59.96 ± 1.61^bc^	60.91 ± 1.17^f^
	T2 (Breaker)	3.97 ± 0.36^h^	56.08 ± 4.58^ab^	58.24 ± 1.97^fg^
	T3 (Pale yellow)	4.86 ± 0.49^de^	55.83 ± 2.80^ef^	53.82 ± 5.76^gh^
	T4 (Dark yellow)	4.50 ± 0.32^de^	58.40 ± 1.32^d^	62.36 ± 1.75^ef^
3L	T1 (Green)	2.95 ± 0.32^f^	56.24 ± 2.72^de^	67.64 ± 3.54^de^
	T2 (Breaker)	3.84 ± 0.06^hi^	59.75 ± 0.64^ab^	71.59 ± 1.02^ab^
	T3 (Pale yellow)	4.56 ± 0.20^ef^	61.13 ± 1.10^ab^	70.54 ± 0.63^bc^
	T4 (Dark yellow)	5.12 ± 0.93^bc^	55.01 ± 2.66^f^	54.46 ± 6.25^h^
*p*		0.03**	0.02**	0.05*

*Note:* Values are expressed as mean ± standard deviation (*n* = 3). For all the values within a column, different letter superscripts mean significant differences (*p* > 0.05) according to Duncan's multiple range test. Fruit maturity stages (T1: Green; T2: Breaker; T3: Pale yellow; and T4: Yellow fruits).

* *p* > 0.05.

** *p* > 0.01.

### 3.2 Variation of Fruit Maturity Stages and Accessions on Proximate Analysis

Variations in moisture content were observed in five different accessions of *M. zeyheri* and four maturity stages at the significant level of *p >* 0.05. Table 2 displays the results, illustrating an increase in moisture content from the T1 (Green) to T4 (Dark yellow) maturity stages in all accessions. Notably, accession HY consistently exhibited the highest moisture content throughout the maturity stages, ranging from 93% to 97%. Accession 6E displayed the highest moisture content at the T2 (Breaker) maturity stage (94%), and accession M7 showed an increase at the T4 (Dark yellow) maturity stage (96%). Moisture content is a key quality indicator, affecting shelf life and suitability for consumption in various forms. The rising moisture content with maturity is due to increased juice content as fruits ripen, as noted by Mahmood et al. (2012).

**TABLE 2:** Nutritional compositions of five accessions of *Mimusops zeyheri* fruits at four fruit maturity stages.

Accessions	Fruit maturity stage	Nutritional compositions (%)			
		Moisture	Dry matter	Ash	Protein
6E	T1 (Green)	93 ± 0.02^e^	91 ± 0.00^a^	4.20 ± 0.00^a^	0.08 ± 0.00^h^
	T2 (Breaker)	94 ± 0.03^d^	89 ± 0.00^cd^	3.94 ± 0.03^b^	0.07 ± 0.00^j^
	T3 (Pale yellow)	94 ± 0.03^d^	89 ± 0.00^ab^	3.75 ± 0.00^d^	0.05 ± 0.00^k^
	T4 (Dark yellow)	95 ± 0.04^c^	90 ± 0.00^ab^	3.42 ± 0.00^g^	0.03 ± 0.00^k^
M7	T1 (Green)	92 ± 0.01^f^	89 ± 0.00^cd^	4.19 ± 0.00^a^	0.17 ± 0.01^e^
	T2 (Breaker)	94 ± 0.03^d^	90 ± 0.00^b^	3.82 ± 0.00^c^	0.07 ± 0.00^hi^
	T3 (Pale yellow)	95 ± 0.04^c^	87 ± 0.00^d^	3.59 ± 0.00^e^	0.06 ± 0.00^i^
	T4 (Dark yellow)	96 ± 0.05^b^	88 ± 0.00^cd^	3.54 ± 0.00^f^	0.04 ± 0.00^j^
3E	T1 (Green)	89 ± 0.00^i^	88 ± 0.02^cd^	3.75 ± 0.00^d^	0.16 ± 0.00^f^
	T2 (Breaker)	90 ± 0.00^h^	88 ± 0.00^cd^	3.26 ± 0.00^i^	0.16 ± 0.00^f^
	T3 (Pale yellow)	91 ± 0.01^g^	87 ± 0.00^de^	3.00 ± 0.00^k^	0.11 ± 0.00^g^
	T4 (Dark yellow)	93 ± 0.02^e^	86 ± 0.00^ef^	3.99 ± 0.00^l^	0.04 ± 0.00^j^
HY	T1 (Green)	93 ± 0.02^e^	90 ± 0.01^abc^	2.97 ± 0.00^l^	0.42 ± 0.00^a^
	T2 (Breaker)	94 ± 0.03^d^	87 ± 0.01^d^	2.81 ± 0.00^m^	0.03 ± 0.00^k^
	T3 (Pale yellow)	96 ± 0.05^b^	89 ± 0.00^ab^	2.78 ± 0.00^n^	0.01 ± 0.00^l^
	T4 (Dark yellow)	97 ± 0.06^a^	90 ± 0.00^b^	2.53 ± 0.00^o^	0.02 ± 0.00^l^
3L	T1 (Green)	91 ± 0.01^g^	87 ± 0.03^d^	3.95 ± 0.00^b^	0.40 ± 0.00^b^
	T2 (Breaker)	93 ± 0.02^e^	88 ± 0.00^de^	3.53 ± 0.00^f^	0.41 ± 0.00^b^
	T3 (Pale yellow)	94 ± 0.03^d^	87 ± 0.00^d^	3.33 ± 0.00^h^	0.31 ± 0.00^c^
	T4 (Dark yellow)	96 ± 0.05^b^	89 ± 0.00^cd^	3.21 ± 0.00^j^	0.20 ± 0.00^d^
*p*		0.00***	0.00***	0.00***	0.00***

*Note:* Values are expressed as mean ± standard deviation (*n* = 3). For all the values within a column, different letter superscripts mean significant differences (*p* > 0.05). Fruit maturity stages (T1: Green; T2: Breaker; T3: Pale yellow; and T4: Yellow fruits).

*** *p* > 0.001.

The ash content of five *M. zeyheri* accessions displayed significant variation (*p >* 0.05) across different maturity stages (Table 2). All accessions showed a decline in ash content from T1 (Green) to T4 (Dark yellow) maturity stages. Accession 6E had the highest ash content at T1 (Green) to T3 (Pale yellow) maturity stage (4.20%–3.75%), while accession 3E had the highest at T4 (Dark yellow) maturity stage (3.99%). Notably, our study's mean ash content values were higher than those reported in previous studies by Mahmood et al. (2012) and Sibiya, Kayitesi, and Moteetee (2021).

Among the selected accessions and maturity stages of *M. zeyheri*, there was significant different (*p >* 0.05) in protein content. Accession HY had the highest protein content at the T1 (Green) maturity stage (0.42%), while accession 3L recorded the highest protein content at T2 (Breaker), T3 (Pale yellow), and T4 (Dark yellow) maturity stages (0.41%–0.20%). The protein content was highest at the T1 (Green) maturity stage and gradually decreased from T2 (Breaker) to T4 (Dark yellow) as the fruits ripened across all accessions, with accession 6E having the least protein content at all maturity stages (0.08%–0.04%). These results align with previous studies on other fruits like jujube ( *Ziziphus mauritiana* Lamk. and *Ziziphus spina‐christi* L.), Loquat ( *Eriobotrya japonica* L.), and Mulberry ( *Morus alba* L.) by Hussain et al. (2010), Nayab et al. (2020), and Amarteifio and Mosase (2006), which also showed a decrease in protein content as fruits matured. This reduction in protein content during fruit maturation is attributed to catabolic processes acting on proteins, as discussed by Diba, Bultosa, and Tolesa (2017). Therefore, the protein content determined in fruits of the selected accessions of *M. zeyheri* demonstrated that they may not regarded as high accumulators of protein.

### 3.3 Variation of Five Accessions and Four Fruit Maturity Stages on Amino Acids Profile

Amino acids profile of five accessions of *M. zeyheri* harvested at different fruit maturity stages are shown in Table 3. Seven out of nine essentials histidine, threonine, lysine, valine, leucine, isoleucine, phenylalanine, and 8 out of the 11 nonessential amino acids arginine, serine, glycine, aspartic acid, glutamine acid, alanine, proline, and tyrosine were determined in this study. The difference in the expression pattern of amino acid metabolizing enzymes occurs during fruit maturity stages process (Boggio et al. 2000). The changes in amino acids content during fruit maturity stages were at most dependent on accessions than fruit maturity (Monti et al. 2016). The content of histidine was increased in accession HY at T1 (Green) and T4 (Dark yellow) maturity stages (0.14 and 0.11 g/100 g), while at T3 (Pale yellow) maturity stage, accession 3E had the highest histidine (0.11 g/100 g). Accession M7 revealed higher histidine at T2 (Breaker) maturity stage of 0.12 g/100 g compared to other accessions (Table 3). According to Dunstan et al. (2017), histidine is used for growth and repair of damaged tissues, including the protection of nerve cells. Threonine was highest in 3L at T1 (Green) and T2 (Breaker) maturity stages (0.21 and 0.24 g/100 g). Threonine is an indispensable amino acid that participate in lipid metabolism and protein synthesis (Edgar 2002). Lysine was undetectable in accessions HY, 3E, M7, and 6E, by contrast, it was detectable in accession 3L at T1 (Green) to T4 (Dark yellow) maturity stages are ranged from 0.02 to 0.07 g/100 g. Mean values of phenylalanine and valine determined during T1 (Green) to T4 (Dark yellow) maturity stages were highest in accessions HY and 3L irrespective of maturity stages. The isoleucine and leucine were detectable at T1 (Green) and T4 (Dark yellow) maturity stages in all accessions whereas accession 3L had highest of isoleucine and leucine at T1 (Green) maturity stage (0.15 g/100 g) (Table 3).

**TABLE 3:** List of essential amino acids of five accessions of *Mimusops zeyheri* fruits at four fruit maturity stages.

Accessions	Fruit maturity stages	Essential amino acids (g/100 g)						
		His	Thr	Lys	Phe	Val	Ile	Leu
6E	T1 (Green)	0.07 ± 0.01^ef^	0.18 ± 0.01^cd^	nd	0.23 ± 0.01^d^	0.12 ± 0.01^e^	0.11 ± 0.01^bc^	0.17 ± 0.01^c^
	T2 (Breaker)	nd	0.17 ± 0.01^def^	nd	nd	nd	nd	nd
	T3 (Pale yellow)	nd	0.17 ± 0.01^def^	nd	nd	nd	nd	nd
	T4 (Dark yellow)	0.08 ± 0.01^e^	0.15 ± 0.01^ef^	nd	0.21 ± 0.01^f^	0.15 ± 0.01^c^	0.07 ± 0.01^f^	0.15 ± 0.01^d^
M7	T1 (Green)	0.13 ± 0.01^b^	0.15 ± 0.01^fg^	nd	0.23 ± 0.01^d^	0.14 ± 0.01^cd^	0.12 ± 0.01^b^	0.21 ± 0.01^b^
	T2 (Breaker)	0.12 ± 0.01^bc^	0.15 ± 0.01^fg^	nd	nd	nd	nd	nd
	T3 (Pale yellow)	nd	0.14 ± 0.01^gh^	nd	nd	nd	nd	nd
	T4 (Dark yellow)	0.10 ± 0.01^cde^	0.12 ± 0.01^h^	nd	0.21 ± 0.01^f^	0.11 ± 0.01^ef^	0.11 ± 0.01^bc^	0.12 ± 0.01^e^
3E	T1 (Green)	0.12 ± 0.01^cde^	0.16 ± 0.01^ef^	0.04 ± 0.01^b^	0.18 ± 0.01^g^	0.12 ± 0.01^e^	0.11 ± 0.01^d^	0.15 ± 0.01^d^
	T2 (Breaker)	0.11 ± 0.01^cde^	0.17 ± 0.01^ef^	nd	nd	nd	nd	nd
	T3 (Pale yellow)	0.11 ± 0.01^cde^	0.18 ± 0.01^def^	nd	nd	nd	nd	nd
	T4 (Dark yellow)	0.08 ± 0.01^e^	0.18 ± 0.01^cd^	nd	0.17 ± 0.01^gh^	0.12 ± 0.01^e^	0.12 ± 0.01^e^	0.18 ± 0.01^c^
HY	T1 (Green)	0.14 ± 0.01^a^	0.12 ± 0.01^h^	nd	0.23 ± 0.01^e^	0.14 ± 0.01^b^	0.08 ± 0.01^e^	0.17 ± 0.01^c^
	T2 (Breaker)	nd	nd	nd	0.24 ± 0.01^bc^	0.16 ± 0.01^b^	nd	nd
	T3 (Pale yellow)	nd	nd	nd	0.24 ± 0.01^bc^	0.16 ± 0.01^b^	nd	nd
	T4 (Dark yellow)	0.11 ± 0.01^cde^	0.21 ± 0.01^b^	nd	0.26 ± 0.01^b^	0.16 ± 0.01^b^	0.11 ± 0.01^d^	0.17 ± 0.01^c^
3L	T1 (Green)	0.11 ± 0.01^cde^	0.24 ± 0.01^a^	0.07 ± 0.01^a^	0.32 ± 0.01^a^	0.21 ± 0.01^a^	0.15 ± 0.01^a^	0.25 ± 0.01^a^
	T2 (Breaker)	nd	0.20 ± 0.01^c^	0.07 ± 0.01^a^	nd	nd	nd	nd
	T3 (Pale yellow)	0.08 ± 0.01^e^	0.15 ± 0.01^fg^	0.04 ± 0.01^b^	nd	nd	nd	nd
	T4 (Dark yellow)	0.08 ± 0.01^e^	0.08 ± 0.01^i^	0.01 ± 0.01^c^	0.17 ± 0.01^h^	0.11 ± 0.01^ef^	0.11 ± 0.01^bc^	0.12 ± 0.01^e^
*p*		0.00***	0.04**	0.12*	0.00***	0.00***	0.00***	0.00***

*Note:* Fruit maturity stages (T1: Green; T2: Breaker; T3: Pale yellow; and T4: Yellow fruits). Histidine (His), threonine (Thr), lysine (Lys), valine (Val), leucine (Leu), isoleucine (Ile), and phenylalanine (Phe). For all the values within a column, different letter superscripts mean ± standard deviation (*n* = 3) significant differences (*p* > 0.05).

Abbreviation: nd, not detected.

* *p* > 0.05.

** *p* > 0.01.

*** *p* > 0.001.

Tyrosine exhibited the highest abundance among amino acids in accession HY when compared to other accessions at maturity stages T1 (Green) to T4 (Dark yellow), with values ranging from 0.28 to 0.41 g/100 g (Table 4). This finding aligns with the role of tyrosine in melanin production, influencing hair and skin color. Accession HY surpassed others in arginine content at T1 (Green) and T4 (Dark yellow) maturity stages (0.19 and 0.18 g/100 g), while accession 3L excelled at T2 (Breaker) and T3 (Pale yellow) maturity stages (0.16 and 0.14 g/100 g), which is relevant to ammonia removal in the body. Serine, glycine, aspartic acid, and glutamine levels significantly increased in accession HY at T4 (Dark yellow) maturity stage, with mean values of 0.13, 0.13, 0.39, and 0.25 g/100 g, respectively. Accession 3L had the highest serine, glycine, and glutamine content at T1 (Green) and T3 (Pale yellow) maturity stages. These results were parallel to those of Kassim, Hambali, and Amir (2017). Alanine content were peaked in accession HY at T1 (Green) maturity stage (0.27 g/100 g), accession 6E at T2 (Breaker) maturity stage (0.18 g/100 g), and accession 3E at T4 (Dark yellow) maturity stage (0.26 g/100 g), but alanine was not detectable in any accessions at T3 (Pale yellow). Proline content was highest in accession 6E at T1 (Green), T2 (Breaker), and T4 (Dark yellow) maturity stages (0.30, 0.29, and 0.25 g/100 g) (Table 4), while accession 3E had the highest value at T3 (Pale yellow) maturity stage (0.19 g/100 g). Proline plays important roles in protein synthesis and structure including the metabolism and nutrition, as well as wound healing, and immune responses (Kassim, Hambali, and Amir 2017).

**TABLE 4:** List of nonessential amino acids of five accessions of *Mimusops zeyheri* fruits at four fruit maturity stages.

Accessions	Fruit maturity stages	Nonessential amino acids (g/100 g)							
		Arg	Ser	Gly	Asp	Glu	Ala	Pro	Tyr
6E	T1 (Green)	0.15 ± 0.01^de^	0.08 ± 0.01^ef^	nd	0.18 ± 0.01^g^	0.17 ± 0.01^ef^	0.34 ± 0.01^a^	0.30 ± 0.01^a^	0.23 ± 0.01^f^
	T2 (Breaker)	0.15 ± 0.01^e^	0.08 ± 0.01^ef^	nd	0.18 ± 0.01^g^	0.17 ± 0.01^ef^	0.18 ± 0.01^c^	0.29 ± 0.01^ab^	0.18 ± 0.01^gh^
	T3 (Pale yellow)	0.12 ± 0.01^f^	Nd	nd	nd	nd	nd	nd	0.18 ± 0.01^gh^
	T4 (Dark yellow)	0.11 ± 0.01^f^	0.08 ± 0.01^ef^	nd	0.21 ± 0.01^f^	0.17 ± 0.01^ef^	0.17 ± 0.01^d^	0.29 ± 0.01^ab^	0.17 ± 0.01^h^
M7	T1 (Green)	0.11 ± 0.01^f^	0.08 ± 0.01^ef^	nd	0.15 ± 0.01^i^	0.15 ± 0.01^fg^	0.12 ± 0.01^f^	0.24 ± 0.01^g^	0.27 ± 0.01^de^
	T2 (Breaker)	Nd	0.08 ± 0.01^ef^	nd	0.17 ± 0.01^h^	0.15 ± 0.01^gh^	nd	nd	nd
	T3 (Pale yellow)	Nd	0.07 ± 0.01^g^	nd	0.17 ± 0.01^h^	0.15 ± 0.01^fg^	nd	nd	nd
	T4 (Dark yellow)	0.12 ± 0.01^f^	0.07 ± 0.01^g^	nd	0.17 ± 0.01^h^	0.14 ± 0.01^i^	0.15 ± 0.01^e^	0.27 ± 0.01^d^	0.28 ± 0.01^d^
3E	T1 (Green)	0.12 ± 0.01^f^	0.08 ± 0.01^ef^	0.08 ± 0.01^f^	0.23 ± 0.01^e^	0.15 ± 0.01^fg^	0.17 ± 0.01^d^	0.18 ± 0.01^hi^	0.17 ± 0.01^h^
	T2 (Breaker)	Nd	Nd	nd	0.23 ± 0.01^e^	nd	nd	0.17 ± 0.01^i^	0.17 ± 0.01^h^
	T3 (Pale yellow)	Nd	Nd	nd	nd	nd	nd	0.18 ± 0.01^hi^	nd
	T4 (Dark yellow)	0.12 ± 0.01^f^	0.08 ± 0.01^ef^	0.11 ± 0.01^e^	0.21 ± 0.01^f^	0.21 ± 0.01^c^	0.18 ± 0.01^c^	0.23 ± 0.01^g^	0.20 ± 0.01^g^
HY	T1 (Green)	0.18 ± 0.01^ab^	0.10 ± 0.01^d^	0.11 ± 0.01^e^	0.36 ± 0.01^b^	0.21 ± 0.01^c^	0.27 ± 0.01^b^	0.26 ± 0.01^e^	0.28 ± 0.01^d^
	T2 (Breaker)	Nd	Nd	nd	nd	nd	nd	nd	0.34 ± 0.01^c^
	T3 (Pale yellow)	Nd	Nd	nd	nd	nd	nd	nd	0.39 ± 0.01^b^
	T4 (Dark yellow)	0.19 ± 0.01^a^	0.14 ± 0.01^b^	0.14 ± 0.01^d^	0.40 ± 0.01^a^	0.26 ± 0.01^b^	0.26 ± 0.01^b^	0.21 ± 0.01^f^	0.41 ± 0.01^a^
3L	T1 (Green)	0.17 ± 0.01^bc^	0.15 ± 0.01^a^	0.21 ± 0.01^a^	0. 32 ± 0.01^c^	0.28 ± 0.01^a^	0.26 ± 0.01^b^	0.28 ± 0.01^c^	0.26 ± 0.01^e^
	T2 (Breaker)	0.17 ± 0.01^cd^	0.12 ± 0.01^c^	0.20 ± 0.01^b^	0. 25 ± 0.01^d^	0.24 ± 0.01^b^	nd	0.26 ± 0.01^e^	0.20 ± 0.01^g^
	T3 (Pale yellow)	0.15 ± 0.01^e^	0.10 ± 0.01^e^	0.17 ± 0.01^c^	0.18 ± 0.01^h^	0.18 ± 0.01^d^	nd	0.19 ± 0.01^h^	0.17 ± 0.01^h^
	T4 (Dark yellow)	0.12 ± 0.01^f^	0.07 ± 0.01^g^	nd	0.12 ± 0.01^k^	0.15 ± 0.01^gh^	0.27 ± 0.01^b^	0.15 ± 0.01^j^	0.15 ± 0.01^i^
*p*		0.001***	0.06*	0.01**	0.001***	0.02**	0.00***	0.00***	0.00***

*Note:* Fruit maturity stages (T1: Green; T2: Breaker; T3: Pale yellow; and T4: Yellow fruits). Arginine (Arg), Serine (Ser), Glycine (Gly), Aspartic acid (Asp), Glutamine acid (Glu), Alanine (Ala), Proline (Pro), and Tyrosine (Tyr). For all the values within a column, different letter superscripts mean ± standard deviation (*n* = 3) significant differences (*p* > 0.05).

Abbreviation: nd, not detected.

* *p* > 0.05.

** *p* > 0.01.

*** *p* > 0.001.

### 3.4 Effect of Accessions and Maturity Stage on Concentration of Macromineral and Micromineral in Fruits of *M*. *zeyheri*

The concentrations of various macronutrient and micronutrient in five accessions of *M. zeyheri*, collected at four fruit maturity stages, displayed significant variation (*p >* 0.05). These nutrients included phosphorus (P), potassium (K), calcium (Ca), magnesium (Mg), sodium (Na), iron (Fe), manganese (Mn), and zinc (Zn). Among the accessions, 3E exhibited the highest P concentration during T1 (Green) to T3 (Pale yellow) maturity stages (9.39, 9.39, and 6.83 mg/L), whereas accession 6E accumulated the highest P during the T4 (Dark yellow) maturity stage (2.73 mg/kg). The P concentrations in this study were lower than those in other published studies, such as Mahmood et al. (2012) on strawberries and Hussain et al. (2010) on pomegranates. Phosphorus is vital for bone and teeth development, metabolism of fats and carbohydrates, and protein production for cell growth and repair (Ndhlala and Tshabalala 2023).

Accession 6E exhibited the highest potassium (K) concentrations at T1 (Green) and T4 (Dark yellow) maturity stages, with values of 50.83 and 44.93 mg/kg, respectively, surpassing all other accessions (Table 5). Conversely, accession HY displayed the lowest K levels at T1 (Green) to T4 (Dark yellow) maturity stages, ranging (38.67–36.40 mg/kg) (Table 5). Notably, the K content in *M. zeyheri* fruits at the T4 (Dark yellow) maturity stage was comparatively lower than that in commonly consumed fruits like bananas (3580 mg/L) and *S. birrea* (averaging 2183 mg/100 g), as reported by Amarteifio and Mosase (2006). Kassim, Hambali, and Amir (2017) noted that during fruit ripening, potassium is utilized to enhance sugar levels, imparting flavor. Potassium plays a vital role in muscle and nerve function, protein synthesis, and overall metabolism, as emphasized by Marieb (2015). According to the (National Academy of Science, Engineering, and Medicine), the recommended daily potassium intake for males aged 2–19 is 2.4 mg/L and for females in the same age group, it is 1.9 mg/L. For adult males aged 20 and above, the recommended intake is 3.016 mg/L, while for adult females, it is 2.320 mg/L (Mitra et al. 2022). Therefore, the fruits of *M. zeyheri* could potentially contribute to addressing potassium deficiency concerns.

**TABLE 5:** List of mineral compositions of five accessions of *Mimusops zeyheri* fruits at four fruit maturity stages.

Accessions	Fruit maturity stages	Macromineral compositions (mg/kg)				
		P	K	Ca	Mg	Na
6E	T1 (Green)	6.44 ± 1.78^bc^	50.83 ± 070^a^	21.80 ± 0.20^b^	4.37 ± 0.12^b^	5.46 ± 2.20^ab^
	T2 (Breaker)	5.50 ± 1.01^bcd^	50.00 ± 783^a^	21.50 ± 0.14^b^	4.25 ± 0.15^b^	5.33 ± 1.36^ab^
	T3 (Pale yellow)	5.40 ± 0.08^bcd^	47.13 ± 0.57^abc^	19.63 ± 0.47^c^	4.01 ± 0.04^c^	3.92 ± 0.34^abcd^
	T4 (Dark yellow)	2.73 ± 1.16^fghi^	44.93 ± 0.28^bcd^	17.17 ± 0.15^e^	3.29 ± 0.09^de^	3.33 ± 0.07^cdefg^
M7	T1 (Green)	4.58 ± 1.32^de^	48.60 ± 0.56^ab^	27.73 ± 0.50^a^	5.96 ± 0.06^a^	5.63 ± 0.60^a^
	T2 (Breaker)	4.47 ± 0.55^def^	45.87 ± 0.40^bc^	18.47 ± 0.32^d^	4.37 ± 0.12^b^	4.72 ± 2.38^abc^
	T3 (Pale yellow)	2.95 ± 2.59^efghi^	45.77 ± 0.12^bc^	17.23 ± 0.57^e^	3.14 ± 0.09^ef^	4.23 ± 1.41^abcd^
	T4 (Dark yellow)	1.19 ± 0.70^ij^	43.93 ± 0.44^cde^	16.47 ± 0.58^ef^	3.13 ± 0.23^efg^	3.64 ± 1.11^bcdef^
3E	T1 (Green)	9.39 ± 0.00^a^	54.97 ± 0.42^bc^	15.60 ± 0.36^g^	2.99 ± 0.13^fg^	5.20 ± 2.20^abc^
	T2 (Breaker)	9.39 ± 0.00^a^	45.60 ± 0.17^bc^	14.63 ± 0.38^h^	2.52 ± 0.02^h^	3.78 ± 0.54^abcdef^
	T3 (Pale yellow)	6.83 ± 3.21^b^	40.70 ± 0.70^ef^	13.93 ± 0.30^hi^	2.40 ± 0.26^hi^	2.47 ± 1.68^defg^
	T4 (Dark yellow)	2.60 ± 0.24^ghi^	39.80 ± 0.11^fg^	11.70 ± 0.62^l^	0.99 ± 0.15^k^	1.74 ± 1.30^g^
HY	T1 (Green)	2.64 ± 0.26^fghi^	38.67 ± 0.35^fg^	14.00 ± 0.44^hi^	2.44 ± 0.05^hi^	2.44 ± 0.19^defg^
	T2 (Breaker)	2.54 ± 0.37^hi^	38.47 ± 0.49^fg^	12.73 ± 0.31^jk^	2.38 ± 0.04^hi^	2.36 ± 0.41^defg^
	T3 (Pale yellow)	2.36 ± 0.30^i^	36.57 ± 0.40^g^	12.93 ± 0.31^jk^	2.33 ± 0.12^i^	1.98 ± 0.12^fg^
	T4 (Dark yellow)	0.19 ± 0.08^j^	36.40 ± 0.46^g^	11.60 ± 0.30^l^	2.05 ± 0.08^j^	1.57 ± 0.32^g^
3L	T1 (Green)	4.89 ± 0.78^cd^	50.73 ± 0.74^a^	16.20 ± 0.20^fg^	3.34 ± 0.09^d^	5.17 ± 1.38^abc^
	T2 (Breaker)	4.38 ± 0.51^defg^	48.70 ± 0.66^a^	15.60 ± 0.36^g^	3.20 ± 0.06^de^	3.78 ± 0.91^abcde^
	T3 (Pale yellow)	4.20 ± 0.51^defgh^	41.70 ± 0.44^def^	13.63 ± 0.96^ij^	2.94 ± 0.22^g^	2.71 ± 0.81^defg^
	T4 (Dark yellow)	2.59 ± 0.46^ghi^	40.20 ± 5.98^efg^	12.27 ± 0.15^kj^	2.46 ± 0.40^hi^	2.13 ± 0.33^efg^
*p*		0.00***	0.00***	0.00***	0.00***	0.001***

*Note:* Values are expressed as mean ± standard deviation (*n* = 3). For all the values within a column, different letter superscripts mean significant differences (*p* > 0.05). Fruit maturity stages (T1: Green; T2: Breaker; T3: Pale yellow; and T4: Yellow fruits). P: Potassium, K: Phosphorus, Ca: Calcium, Mg: Magnesium, Na: Sodium.

*** *p* > 0.001.

Accession M7 exhibited the highest mean Ca (27.73 mg/kg) at T1 (Green) maturity, whereas accession 6E showed increased Ca levels at T2 (Breaker) and T3 (Pale yellow) maturity stages (21.50 and 17.17 mg/kg), differing from other accessions (Table 5). This indicates a decreasing trend in Ca concentration from T1 (Green) to T4 (Dark yellow), consistent with Chukwuka, Iwuagwu, and Uka (2013). Accession HY had the lowest Ca (11.60 mg/kg) at T1 (Green) maturity. Calcium is crucial for bone and teeth development. Fruit Ca content affects quality, cell structure, enzyme activity, and ripening, influencing fruit firmness and storability. Consuming indigenous fruits may mitigate mineral deficiencies.

Accession M7 exhibited superior performance compared to other accessions during the T1 (Green) and T2 (Breaker) maturity stages, displaying the highest concentrations of Mg at 5.96 and 4.37 mg/kg, respectively. Following closely was accession 6E, which recorded significant Mg concentrations at T3 (Pale yellow) and T4 (Dark yellow) maturity stages (4.01 and 3.29 mg/kg). Magnesium plays a pivotal role in various physiological processes, such as protein synthesis, bone formation, DNA replication, blood pressure regulation, blood sugar control, and neuromuscular function (Sojinu et al. 2021). Accession M7 also displayed notably elevated Na concentrations at T1 (Green), T2 (Breaker), and T4 (Dark yellow) maturity stages (5.63, 4.23, and 3.64 mg/kg). Accession 6E had the highest mean value of 5.33 mg/kg at the T4 (Dark yellow) maturity stage (Table 5).

The micronutrients, accession 6E exhibited the highest Fe accumulation at the T1 (Green) to T3 (Pale yellow) maturity stages, with concentrations of 1.57, 0.62, and 0.37 mg/kg, surpassing other accessions (Table 6). Similar findings of elevated iron content in fruits have been documented in previous scholarly studies, such as the research conducted by Amarteifio and Mosase in 2006. Conversely, accession 3E demonstrated an increase in iron content at the T4 (Dark yellow) maturity stage, reaching 0.49 mg/kg. If the iron present in these fruits were 100% bioavailable, daily consumption of 386.3 g of early stage or 300 g of late‐stage fruits would be required to attain 18 mg of iron. For pregnant women, this would equate to 579 or 450 g of early stage or late‐stage fruits per day, as suggested by Diba, Bultosa, and Tolesa (2017). Despite being a micronutrient, iron is essential for various physiological functions in the human body, including growth, hemoglobin production, and protein synthesis in red blood cells, which transport oxygen from the lungs to all body tissues, as highlighted in the study by Sibiya, Kayitesi, and Moteetee (2021). Additionally, iron plays a crucial role in facilitating the regulation of body weight by proteins, carbohydrates, and fats, making it a significant factor in the management of conditions such as diabetes, as observed in the research by Moses et al. (2012).

**TABLE 6:** List of mineral compositions of five accessions of *Mimusops zeyheri* fruits at four fruit maturity stages.

Accessions	Fruit maturity stages	Micromineral compositions (Mg/kg)		
		Fe	Mn	Zn
6E	T1 (Green)	1.57 ± 0.07^a^	0.22 ± 0.01^a^	0.66 ± 0.03^c^
	T2 (Breaker)	0.62 ± 0.16^bc^	0.20 ± 0.01^ab^	0.6 ± 0.03^c^
	T3 (Pale yellow)	0.40 ± 0.01^efgh^	0.17 ± 0.01^bc^	0.18 ± 0.10^ef^
	T4 (Dark yellow)	0.37 ± 0.04^fghi^	0.14 ± 0.01^d^	0.41 ± 0.20^d^
M7	T1 (Green)	0.66 ± 0.01^b^	0.19 ± 0.01^abc^	0.38 ± 0. 09^d^
	T2 (Breaker)	0.48 ± 0.13^def^	0.11 ± 0.01^de^	0.17 ± 0. 04^ef^
	T3 (Pale yellow)	0.47 ± 0.07^def^	0.09 ± 0.03^fg^	0.16 ± 0.18^f^
	T4 (Dark yellow)	0.44 ± 0.04^defg^	0.08 ± 0.02^fg^	0.06 ± 0.18^g^
3E	T1 (Green)	0.55 ± 0.06^cd^	0.18 ± 0.01^bc^	1.73 ± 0.19^a^
	T2 (Breaker)	0.51 ± 0.15^cd^	0.09 ± 0. 03^ef^	1.10 ± 0.00^b^
	T3 (Pale yellow)	0.49 ± 0.07^def^	0.01 ± 0.01^k^	0.60 ± 0.20^c^
	T4 (Dark yellow)	0.28 ± 0.02^ij^	0.01 ± 0.01 k	0.15 ± 0.06^f^
HY	T1 (Green)	0.37 ± 0.02^ghij^	0.08 ± 0.01^fgh^	0.27 ± 0.09^h^
	T2 (Breaker)	0.30 ± 0.04^hij^	0.07 ± 0.01^fgh^	0.16 ± 0.03^ef^
	T3 (Pale yellow)	0.25 ± 0.01^j^	0.06 ± 0.03^fgh^	0.13 ± 0.10^f^
	T4 (Dark yellow)	0.24 ± 0.06^j^	0.06 ± 0.01^fgh^	0.05 ± 0.04^g^
3L	T1 (Green)	0.51 ± 0.07^de^	0.07 ± 0.01^ghi^	0.34 ± 0.14^de^
	T2 (Breaker)	0.49 ± 0.05^de^	0.06 ± 0.01^hi^	0.24 ± 0.04^def^
	T3 (Pale yellow)	0.35 ± 0.02^ghij^	0.04 ± 0.01^ij^	0.24 ± 0.07^def^
	T4 (Dark yellow)	0.33 ± 0.02^ghij^	0.02 ± 0.00^jk^	0.17 ± 0.09^ef^
*p*		0.00***	0.00***	0.00***

*Note:* Values are expressed as mean ± standard deviation (*n* = 3). For all the values within a column, different letter superscripts mean significant differences (*p* > 0.05). Fruit maturity stages (T1: Green; T2: Breaker; T3: Pale yellow; and T4: Yellow fruits). Fe: Iron, Mn: Manganese, Zn: Zinc.

*** *p* > 0.00.

The manganese (Mn) concentration in accession 6E significantly increased from 0.22 mg/kg at T1 (Green) to 0.14 mg/kg at T3 (Pale yellow) maturity stages, whereas accession 3L had the lowest Mn levels at T1 (Green) 0.07 mg/kg and T2 (Breaker) 0.06 mg/kg. Accession 3E displayed similar Mn values (0.01 mg/L) at T3 (Pale yellow) and T4 (Dark yellow) maturity stages. Manganese is crucial for normal brain function (Marieb 2015). Accession 3E had the highest zinc (Zn) levels at T1 (Green) to T3, (Pale yellow) declining at T4 (Dark yellow) maturity stages, while accession 6E exhibited increased Zn at T4 (Dark yellow) maturity stage. Zinc is essential for growth, immunity, DNA synthesis, and various cellular functions (Khawas and Deka 2016; Gudes et al. 2017). *M. zeyheri* fruits contribute modestly to recommended dietary Zn intake (8–12 mg) per day.

## 4 Conclusion

This investigation affirms that variations in quality, physicochemical properties, and nutritional content findings underscore the potential for cultivating and marketing *M. zeyheri* fruits as fresh snacks or freeze‐dried powder for food supplementation programs. Moreover, the study revealed detectable levels of nutritional, amino acid, and mineral contents across all five accessions, with significant variations based on accession and maturity stage. While the size of *M. zeyheri* fruits did not change significantly during the transition from green to yellow, their TSS increased, and the TSS/TA ratio rose, suggesting ripeness. Fruit firmness consistently decreased as fruits ripened. This changes were in concomitant to an increase in moisture content and a decrease in ash and protein content. Accessions M7 T1–T4 exhibited the highest essential amino acids including histidine and threonine, as well as Ca, Mg, and Na. Accessions 3E and 6E at T2 and T3 maturity stage exhibited the highest P, Fe, Zn, and Mn.

Promoting *M. zeyheri* aligns with the UN Sustainable Development Goals 2030. Furthermore, these fruits can offer an accessible, abundant, and cost‐effective source of functional food with substantial health advantages, with accessions 3E, 6E, and M7 and HY recommended at the T2 to T3 fruit maturity stage. In conclusion, it is essential to foster awareness and utilization of *M. zeyheri* as a valuable food source, while also encouraging further research into its cultivation and potential health benefits. By doing so, we can enhance the livelihoods of rural communities and contribute to global efforts in combating malnutrition and promoting food security.

## Author Contributions

**Kamogelo P. Teffo:** data curation (equal), formal analysis (equal). **Semakaleng Mpai:** conceptualization (equal), writing, and editing (review), data curation (equal), investigation (equal), methodology (equal), supervision (supporting). **Ashwell R. Ndhlala:** conceptualization (lead), funding acquisition (lead), investigation (lead), project administration (equal), resources (equal), software (equal), supervision (equal), writing – review and editing (equal). **Phatu W. Mashela:** conceptualization (equal), data curation (equal), project administration (equal).

## Conflicts of Interest

The authors declare no conflicts of interest.

## Data Availability

Data is available on request.
